# Impact of nutritional and educational support on home enteral nutrition

**DOI:** 10.1186/s41043-023-00384-4

**Published:** 2023-05-22

**Authors:** Adela Madrid-Paredes, Socorro Leyva-Martínez, Verónica Ávila-Rubio, Juan Bautista Molina-Soria, Patricia Sorribes-Carrera, Carmen Yeste-Doblas, José Antonio López-Medina, Victoria Eugenia Luna-López, María Luisa Fernández-Soto

**Affiliations:** 1 Servicio Farmacia Hospitalaria, Hospital Universitario San Cecilio, Instituto de Investigación Biosanitaria de Granada, Av. del Conocimiento, s/n, 18016 Granada, Spain; 2 Unidad de Nutrición Clínica y Dietética, Hospital Universitario San Cecilio, Instituto de Investigación Biosanitaria de Granada, Av. del Conocimiento, s/n, 18016 Granada, Spain; 3 Unidad de Nutrición Clínica y Dietética, Hospital Universitario San Agustín, AGS Norte de Jaén en Linares (Jaén), Av. San Cristóbal, 2D, 23700 Linares, Jaén, Spain; 4 Unidad de Nutrición Clínica y Dietética, Consorcio Hospitalario Provincial de Castellón, Avda. Dr. Clará 19 (704,29 km), 12002 Castellón de La Plana, Spain; 5 Unidad de Nutrición Clínica y Dietética, Hospital Universitario Virgen de la Victoria, Campus de Teatinos, s/n, 29010 Málaga, Spain; 6 Unidad de Nutrición Clínica y Dietética, Hospital Universitario Virgen de las Nieves, Av. de las Fuerzas Armadas, 2, 18014 Granada, Spain; 7 Departamento de Medicina, Facultad de Medicina, Av. de la Investigación, 11, 18016 Granada, Spain

**Keywords:** Enteral, Home, Nutrition, Education

## Abstract

**Background:**

Home Enteral Nutrition (HEN) is used to prevent or correct malnutrition in outpatients. Due to the complexity of this process, the indication, follow-up, and results of an educational program of HEN patients was evaluated.

**Methods:**

A prospective, observational, real-life, multicenter study was performed in 21 Spanish Hospital. Patients receiving HEN by nasogastric tube or ostomy were included. The following variables were collected: age, gender, HEN indication, type of formula, nutritional requirements, laboratory variables, complications, and quality standards of the educational program. To calculate the energy and protein requirements, the FAO/WHO/UNU formula was used considering the adjusted weight of the patients. All data were analyzed using SPSS.24.

**Results:**

414 patients were included. Most conditions diagnosed were neurodegenerative diseases (64.8%). 100 (25.3%) were diabetic. The mean weight was 59.3 ± 10.4 kg and BMI 22.6 ± 3.2. Moderate protein-calorie malnutrition was predominant at baseline (46.4%). Improvement in nutritional status at six months was recorded in more than 75% of patients (*p* < 0.05). Tolerance problems, diarrhea and abdominal distension fell between the 3- and 6-month visits (*p* < 0.05). Patients who received intermittent EN had fewer tolerance-related effects (OR 0.042; 95% CI 0.006–0.279) and less diarrhoea (OR 0.042; 95% CI 0.006–0.279). At the baseline and 6-month visits, compliance with the educational measures proposed by the prescriber was ≥ 99%.

**Conclusion:**

The nutritional assessment to prescribe individualized HEN to each patient, together with educational measures and training in the proper use of this treatment for both patients and trainers, improves nutritional status and reduces the onset of adverse events.

**Supplementary Information:**

The online version contains supplementary material available at 10.1186/s41043-023-00384-4.

## Background

Home Enteral Nutrition (HEN) or the administration of enteral formula via the digestive tract, usually by tube, is used to prevent or correct malnutrition in patients cared for at home [1]. HEN is a type of nutritional support that is being increasingly prescribed. In the US, the population receiving HEN in 1992 was 415 patients per million inhabitants [2], which had increased to 1,385 patients per million population by 2013 [3]. This therapy brings a number of benefits to patients, their families and the healthcare network that provides it [4]. It entails a reduction in costs, with an estimated saving of 75% with the uptake of home treatment [5].

One of the objectives of healthcare professionals is to ensure that patients are monitored at home and that the established treatment is correctly maintained, as well as to control and minimize possible side effects, to ensure the therapeutic goals are met [6]. For patients receiving HEN, it is important to remember the importance of ensuring adequate nutrient intake, as malnutrition is associated with increased morbidity and risk of complications in a wide range of patients. This includes patients with chronic obstructive disease, post-stroke patients or bedridden patients at increased risk of pressure ulcers [7, 8], and surgical patients (pre- and post-operative) [9], but especially multimorbid and chronic patients where the incidence of the population with swallowing difficulty primarily due to neurodegenerative problems is very high [10]. The selection of the most appropriate formula for each patient has been shown to achieve both energy and nutrient therapeutic goals [11] and improve nutritional status.

Adequate patient and/or family training by a qualified professional is necessary for HEN to be feasible [6, 12, 13]. This process was hampered during the COVID-19 pandemic and could influence adherence/compliance to treatment where telemedicine became an essential tool to support these patients [14].

In terms of patient monitoring and follow-up, the coordination of a multidisciplinary team is essential to prevent complications such as malnutrition and dehydration in these patients [8], as well as complications caused by the EN itself, such as vomiting, diarrhea, constipation, abdominal distension, and complications arising from the access route (nasogastric tube [NGT], percutaneous endoscopic gastrostomy [PEG] and percutaneous radiological gastrostomy [PRG]), such as obstruction [15].

Due to the complexity of this process and the high impact that the initiation of these treatments and the associated complications can have on the Spanish National Health System (NHS), it was considered necessary to analyze data on the indication, treatment and follow-up of a group of patients included in the HEN.1 study, as well as the results of an educational program, with the main objective of improving quality of care of the HEN process and optimizing the use of this resource within the Spanish NHS.

## Methods

### Study design

A prospective, observational, multicenter real-life study was conducted.

### Study population

All patients 18 years of age and over prescribed HEN treatment with nasogastric tube or PEG by the corresponding Clinical Nutrition Unit of 21 hospitals, who maintained their treatment at home or in a residence from 1 July 2016 to 1 July 2020, with stabilized underlying disease, who had accepted and signed the informed consent (patient or caregivers) and who fulfilled a series of the following clinical indications were included:

- Mechanical impairment of swallowing or transit with severe aphagia or dysphagia requiring a feeding tube and/or PEG.
- Neuromotor disorders that prevent swallowing or transit requiring a feeding tube and/or PEG.
- Patients with special energy and/or nutrient requirements requiring a feeding tube and/or PEG.

The exclusion criteria were as follows: pregnant women, expected survival under 180 days, unstable patients, and a failure to sign the informed consent for any reason. In older patients with a scarce social and family environment, the educational program has been reinforced, and the health area nurse has been informed for further follow-up. This study was approved by the CHUG Ethics and Research Committee. The STROBE-nut checklist [16] was filled (Additional file 1: Table S1).

### Clinical, biochemical and nutritional variables

The variables were collected at face-to-face, telephone and/or home visits (baseline, 3 and 6 months). At the initial visit, the following variables were collected: age, gender, comorbidity, diagnosis that led to the indication for HEN (neurological disease, neoplastic disease, stroke) and presence of malnutrition, and a nutritional assessment was performed. To calculate energy and protein requirements, the FAO/WHO/UNU formula was used considering the adjusted weight of the patients.

Parameters concerning the initiation and adequacy of HEN support according to the needs and clinical course of the patients were collected: calculation of calorie-protein requirements, type of polymeric EN formula (hyperproteic/hypercaloric [HP/HC], normoproteic/normocaloric [NP/NC] and diabetic), volume of nutritional formula, administration regimen, access route, average duration of use of enteral support and HEN-related complications. All formulas used were complete polymeric formulas. A complete hyperproteic/hypercaloric polymeric formula was defined when its protein content was greater than 18% of the total caloric value (TCV) and the caloric density greater than 1.30 kcal/ml. The complete normoprotein/normocaloric formula was defined when the protein content was equal or less than 18% of the TCV and the caloric density was between 0.9 and 1.10 kcal/ml. A diabetic diet was considered as a special polymeric formula characterized by lipids with a high content of monounsaturated fatty acids (FA), carbohydrates with low glycemic indices and soluble fiber.

The type of calorie, protein and protein-calorie malnutrition was determined according to the criteria of the "SENPE-SEDOM Document on the coding of hospital malnutrition" [17].

The following anthropometric and laboratory variables were collected at baseline and at 6 months: height (m), usual weight (kg), current weight (kg), BMI (kg/m2), percentage weight loss (%), mid-upper arm circumference (MUAC) (cm), glucose (mg/dl), albumin (mg/dl), prealbumin (mg/dl), transferrin (mg/dl), total cholesterol (mg/dl), HDL cholesterol (mg/dl), LDL cholesterol (mg/dl), triglycerides (mg/dl) and lymphocytes (mg/dl). Patients who experienced one or more problems (nausea, vomiting, diarrhea, constipation, abdominal distension, and regurgitation) were considered to have tolerance issues. Improved nutritional status was also assessed in terms of a reduction in severity of energy or protein malnutrition (severe to moderate, moderate to mild or mild to well nourished) and with changes in anthropometric and biochemical variables between the initial visit and at 6 months.

### Educational programme compliance variables

An educational program was prepared with an initial session on the main complications of the feeding tube, delivery of written information and subsequent regular telephone sessions. It was recorded whether the clinical report and educational material had been delivered, if it had been adequately explained to the patient how to manage and maintain the diet, and the access route details, as well as any interim or final suspensions.

### Statistical analysis

The data obtained were entered into an Access database®, available by contacting the authors. Statistics of central tendency were used to describe the variables (mean, standard deviation) because tests of normality of the data indicated that the data are normally distributed (Kolmogorov–Smirnov and Shapiro–Wilk tests). Qualitative variables were described by frequency and proportion. To assess any significant differences during the treatment period (baseline visit and 6-month visit), a paired mean difference (t-test for related data) or a Chi-square test (or Fisher's exact test when the conditions for applying the chi-square were not met) and McNemar's test were used as appropriate. If three periods were evaluated (baseline, 3 months and 6 months), an ANOVA was performed for repeated measures. Finally, a logistic regression model was used to identify the risk factors related to tolerance and adverse effects of supplementation. The significance level used was 5% (*p*-value < 0.05). All data were analyzed using version 24 of the SPSS statistical software.

## Results

### Study population and prescribed enteral nutrition formulas

In total, 414 patients were included, 254/414 (61.4%) of whom were women. The most prescribed EN formula was hyperproteic/hypercaloric without fiber (145/414; 35.0%). The main conditions diagnosed were neurodegenerative disease (Alzheimer's, Parkinson's, dementia) in 64.8% of cases (*n* = 256), and stroke in 22.8% of cases (*n* = 90). One hundred patients (25.3%) of the total sample were diagnosed with diabetes mellitus. The patients had a mean weight of 59.3 ± 10.4 kg and a BMI of 22.6 ± 3.2 kg/m^2^ (Table 1). The prevalence of the female population was higher in stroke (59/91; 64.8%) and neurodegenerative disease (178/259; 68.7%) compared to head and neck cancer patients, in which the prevalence of male population was higher (37/45; 82.2%). The prescription of an HP/HC formula with fiber was higher in head and neck cancer patients (12/45; 26.6%) and neurodegenerative disease (54/258; 21.0%) compared to patients with stroke (11/91; 12.1%) (*p* < 0.001). However, the prescription of an NP/NC formula with fiber was higher in patients with stroke (23/91; 25.2%) and neurodegenerative disease (54/258; 20.9%) compared to head and neck cancer patients (1/45; 2.2%) (*p* < 0.001) (Table 2).

**Table 1:** Description of the population and type of enteral formula prescribed at the baseline visit

		*N* total	*n* (%)/ n ± (SD)
Age (years)		414	77.9 ± 12.1
Gender (M/F)			160 (38.6)/ 254 (61.4)
HP/HC formula with fibre		414	77 (18.6)
HP/HC formula without fibre		414	145 (35)
NP/NC formula with fibre		414	78 (18.8)
NP/NC formula without fibre		414	47 (11.4)
Diabetic formula		414	67 (16.2)
Clinical condition	Stroke		90 (22.8)
	Neurodegenerative disease		256 (64.8)
	Head and neck cancer		42 (10.6)
Diabetes Mellitus		395	100 (25.3)
Baseline weight (kg)		391	59.3 ± 10.4
Baseline BMI (kg/m^2^)		392	22.6 ± 3.2
Baseline MUAC (cm)		72	25.6 ± 5.7

*F* Female, *HC* Hypercaloric, *HP* Hyperproteic, *M* Male, *N* Population, *NC* Normocaloric, *NP* Normoproteic, *MUAC* Mid-upper arm circumference

**Table 2:** Description of the population according to diagnosed clinical condition

	Clinical condition						*p*-value
	Stroke		Neurodegenerative disease		Head and neck cancer		
	*N*	*n* (%)	*N*	*n* (%)	*N*	*n* (%)	
Sexo (women)	91	59 (64.8)	259	178 (68.7)	45	8 (17.8)	0.000
HP/HC formula with fiber	91	11 (12.1)	258	54 (21.0)	45	12 (26.6)	0.000
HP/HC formula without fiber	91	26 (28.5)	258	103 (39.9)	45	16 (35.5)	0.873
NP/NC formula with fiber	91	23 (25.2)	258	54 (20.9)	45	1 (2.2)	0.000
NP/NC formula without fiber	91	7 (7.7)	257	34 (13.2)	45	6 (13.3)	0.156
Diabétic formula	91	14 (15.4)	259	47 (18.1)	45	6 (13.3)	0.657

*HC* Hypercaloric, *HP* Hyperproteic, *N* Sample, *NC* Normocaloric, *NP* Normoproteic

### Nutritional status

At baseline, moderate protein-calorie malnutrition was predominant among the patients recruited for the study (*n* = 192, 46.4%). The change at 6 months showed a significant improvement in nutritional status (Table 3).

**Table 3:** Nutritional status of the study population at baseline and 6 months visit

Visit			
	Baseline	6 months	*p*-value
	*n* (%)	*n* (%)	
Mild calorie malnutrition	26 (6.3)	40 (9.9)	0.144
Moderate calorie malnutrition	19 (4.6)	4 (1.0)	0.000
Severe calorie malnutrition	2 (0.5)	0 (0.0)	0.885
Mild protein malnutrition	29 (7.0)	49 (12.1)	0.000
Moderate protein malnutrition	31 (7.5)	3 (0.7)	0.022
Mild protein-calorie malnutrition	58 (14)	169 (41.8)	0.000
Moderate protein-calorie malnutrition	192 (46.4)	76 (18.8)	0.000
Severe protein-calorie malnutrition	22 (5.3)	4 (1.0)	0.035
Well nourished	35 (8.5)	59 (14.6)	0.012
n: sample			

According to the changes that occurred between one period and another, nutritional status improved in more than 75% of patients (Table 4).

**Table 4:** Improvement in nutritional status by diagnosed clinical condition

	Improved nutritional status		
		YES/NO	%
Clinical condition	Stroke	75/90	83.3
	Neurodegenerative disease	202/256	78.9
	Head and Neck Cancer	39/42	92.9

### Biochemical parameters

For the variables glucose, albumin, prealbumin, lymphocytes, transferrin, weight, body mass index (BMI) and mid-upper arm circumference (MUAC), an improvement was observed between baseline and 6 months (*p*-value < 0.05) (Table 5).

**Table 5:** Biochemical parameters at baseline and 6-months visit

	Baseline		6-months		
	*n*	Mean ± SD	*n*	Mean ± SD	*p*-value
Glucose (mg/dl)	208	114.0 ± 41.0	181	106.0 ± 30.0	0.006
Total cholesterol (mg/dl)	171	165.0 ± 34.0	142	167.0 ± 38.0	0.189
LDL cholesterol (mg/dl)	101	111.8 ± 33.1	73	107.0 ± 33.0	0.421
HDL cholesterol (mg/dl)	101	40.0 ± 21.0	71	40.0 ± 23.0	0.964
Triglycerides (mg/dl)	163	121.0 ± 45.0	135	126.0 ± 96.0	0.461
Albumin (mg/dl)	184	3.0 ± 0.6	166	3.56 ± 0.44	0.000
Prealbumin (mg/dl)	60	15.13 ± 5.47	57	21.1 ± 3.9	0.000
Lymphocytes (cell/mm^3^)	166	1.61 ± 0.69	145	1.79 ± 0.82	0.010
Transferrin (mg/dl)	35	206.0 ± 47.0	28	234.0 ± 62.0	0.050
Weight (kg)	391	59.3 ± 10.4	383	60.4 ± 9.3	0.000
BMI (kg/m^2^)	392	22.66 ± 3.22	381	23.1 ± 2.8	0.000
MUAC (cm)	72	25.6 ± 5.8	57	27.1 ± 6.0	0.000

*n*: sample, *SD* Standard deviation

### Adverse effects

Adverse effects were recorded in no more than 6% of cases, except for tolerance problems, where the percentage was 16.3%. There was a reduction in all adverse effects between the 3-month and 6-month visits, with significant reductions observed for tolerance problems, diarrhea and abdominal distension (Table 6).

**Table 6:** Tolerance problems at 3 and 6 months visit

	3-months	6-months	*p*-value
	*n* (%)	*n* (%)	
Tolerance problems	67 (16.3)	20 (4.9)	< 0.001
Diarrhoea	23 (5.6)	5 (1.2)	< 0.001
Constipation	11 (2.7)	6 (1.4)	0.302
Abdominal distension	21 (5.1)	2 (0.5)	< 0.001
Nausea	7 (1.7)	1 (0.2)	0.031
Vomiting	8 (1.9)	0 (0.0)	–
Regurgitation (reflux)	9 (2.2)	2 (0.5)	0.065
Fever	0 (0.0)	1 (0.2)	–

### Administration route and adverse effects

At 6 months there was a slight increase in NGT administration (58.3% to 63.2%) to the detriment of PEG (41.7% to 36.8%). When analyzing the association between the occurrence of adverse reactions and the access route, a higher occurrence of diarrhea was observed in patients with NGT vs PEG at 3 months (19/239; 7.9% vs 4/171; 2.3%; *p* = 0.015). The most commonly used administration regimen was intermittent in 399/412 patients (96.8%). Patients with intermittent administration had lower tolerance and diarrhea problems than patients with continuous administration at 6 months (4.3% vs 23.1%; *p* = 0.021 and 0.8% vs. 15.4%; *p* = 0.009, respectively). The most commonly used feedings/day regimens were 4 or 5 per day (41.1% *n* = 156 and 53.7% *n* = 204, respectively). When the association between the occurrence of abdominal distension and feedings/day at 3 months was analyzed, it was found that the higher the number of feedings per day, the lower the occurrence of constipation (*p* = 0.039). In the case of abdominal distension, the 5 feedings/day regimen was associated with greater nausea (*p* = 0.001). Patients who received intermittent EN had fewer tolerance-related effects in general (OR 0.042; 95% CI 0.006–0.279) and diarrhea (OR 0.042; 95% CI 0.006–0.279), while those who received continuous EN.

### Nutritional requirements

The established energy and protein requirements remained constant throughout the follow-up of the patients in the study. The mean energy requirements were 1548 ± 297, 1465 ± 295 and 1550 ± 291 kcal/day at baseline, 3 months and 6 months, respectively. Protein requirements were also very constant over the three visits; 70.7 ± 15, 70.5 ± 15.7 and 70.8 ± 15 g/day, respectively. In relation to the requirements by condition described, both protein and energy requirements were found to be higher in patients diagnosed with neoplastic disease than for the other conditions (*p*-value < 0.05) (Additional file 2: Table S2). When analyzing the relationship between the onset of adverse reactions at 3 months with the need to adjust nutritional requirements, it was observed that these differences were significant for abdominal distension (*p* < 0.001) and nausea (*p* = 0.017). This statistical significance was maintained for abdominal distension at 6 months (*p* < 0.001).

### Educational programme compliance

At the baseline visit, compliance with the measures proposed by the prescriber was: delivery of clinical report (392; 100%), delivery of educational material (392; 100%), correct adherence to the diet (383; 100%), indications for access route (391; 100%) and proper handling of the diet (407; 99.3%), and at 6 months, correct adherence to the diet (387; 99.7%), indications for access route (382; 99.2%) and proper handling of the diet (381; 99.0%). There were very few interim and final suspensions (1; 0.3% and 2; 0.5%, respectively) in the 6-month review. However, 20% of patients required an adjustment to their nutritional regimen for various reasons. From the baseline to the 3-month visit, of all patients who had tolerance problems, almost 40% (39.4%, *n* = 66) were offered an adjustment to requirements and/or feeding regimens (*p*-value < 0.001), and between the 3-month and 6-month visit, 40% (*n* = 20) of patients who had tolerance problems were also offered an adjustment to requirements and/or feeding regimens (*p*-value < 0.001).

## Discussion

HEN may improve the nutritional status and quality of life of patients and their families. Healthcare professionals must therefore ensure that the prescribed formulas are appropriate for each individual patient and that nutritional requirements are adjusted on a case-by-case basis. Patient and family training for the proper use of preparations and monitoring at home is key to ensuring correct adherence to the established treatment, controlling and minimizing possible side effects and thereby ensuring the therapeutic goals are met. This study assessed the 6-month evolution of the prescribed nutritional requirements, nutritional status, adverse effects and compliance with the proposed regimens for the correct use of EN in 414 patients included in the HEN.1 study.

HEN led to an improvement in nutritional status in more than 75% of patients across all conditions, with adverse effect rates of less than 5%, except for tolerance problems, which were 16.3%.

In our study, the most common reason for indicating EN was neurodegenerative diseases (64.5%), consistent with the Spanish HEN registry (NADYA) (59%) [18]. However, the average age of our population was somewhat higher at 78 vs 71 years [18], perhaps because neurodegenerative disease, the most frequent reason for prescribing HEN in both this study and in the registry, is more common in elderly people. Cancer patients had a higher use of HP/HC formulas with fiber (26.6%) than the rest of the conditions (*p* < 0.001) (Table 2). This was due to their higher energy and protein requirements (Additional file 2: Table S2), in line with the recommendations of the European guidelines on nutrition in cancer patients [19], which specify energy requirements of 25–30 kcal/kg/day and a high protein intake, as this promotes muscle protein anabolism in cancer patients [20].

At 6 months there was a statistically significant improvement in nutritional status (Table 3) and more than 75% across all conditions (Table 4). This improvement in nutritional status was also reflected in the evolution of the laboratory parameters, with a statistically significant improvement between baseline and 6 months (Table 5). These findings are consistent with a study of 102 patients (56.9% with HEN and 43.1% with hospital EN) treated with a hyperproteic/hypercaloric formula for 8 weeks, which also found improved weight, BMI, albumin and prealbumin parameters [21].

In a systematic review of elderly people with dementia and tube feeding, no improvement in nutritional status was seen [22]. However, PEG placement in patients with dysphagia resulted in a significant increase in caloric intake by 30.5%, protein intake by 26.0% and a conversion of the protein balance from negative to neutral in a study of 117 patients in a geriatric center [23]. For cancer patients, a recent meta-analysis that included 1059 patients with upper gastrointestinal tract cancer showed that HEN also reduced the incidence of malnutrition and latent malnutrition (RR: 0.54; *p* < 0.01) [24].

The adverse effects experienced by the patients in our study did not exceed 6% of cases, except for tolerance problems, which was 16.3% at 3 months. In all cases, a favorable clinical course was observed from one visit to the next, with a statistically significant reduction in gastrointestinal symptoms at the 3- and 6-month visits in terms of tolerance problems, diarrhea and abdominal distension (Table 6). These findings are consistent with the Ballesteros et al. study in which gastrointestinal adverse reactions were also reduced (nausea, regurgitation, constipation, diarrhea, flatulence and distension), *p* < 0.05 [25]. A factor that may have affected the good tolerability of HEN may be that 96.8% of our patients had intermittent EN instead of continuous administration. In the CAFANE study in which HEN was administered to 304 patients, intermittent EN use was a protective factor against vomiting (OR 0.4; *p* = 0.037), regurgitation (OR 0.3; *p* = 0.002), constipation (OR 0.3; *p* = 0.000), diarrhea (OR 0.4; *p* = 0.007) and abdominal distension (OR 0.4; *p* = 0.006) compared with bolus administration [15]. Our results were similar, as intermittent EN administration was also a protective factor against tolerance problems (OR 0.042; *p* = 0.001) and diarrhea (OR 0.042; *p* = 0.001), confirming that whenever possible, EN should be administered with this regimen.

At 6 months, only one interim suspension (1/390; 0.3%) and two final suspensions (2/390; 0.5%) occurred from all included patients who had received EN via NGT or PEG. This fact, together with the low incidence of reported adverse reactions, suggests that overall tolerance must have been good, as was the case in a cohort of 51 patients with HEN treated with a hyperproteic/hypercaloric formula [26].

Prescriber compliance was recorded for some proposed measures such as quality indices of the service provided and patient/caregiver educational measures. Compliance > 99% was obtained for all measures, both at the baseline and 6-month visits. Up to 68% of NGT patients did not comply with the prescribed HEN programs in a study of head and neck cancer patients in which 36/88 had PEG [27]. Moreover, an average of 5.4 unscheduled medical care contacts were necessary to resolve the complications experienced by 8 patients with HEN over a mean follow-up time of 10.5 months [6]. The technical training of patients/caregivers for correct EN administration and to resolve any equipment- and tube-related problems is essential for optimizing treatment [1, 28].

The most important limitation of our study is that it is a prospective, observational design with no control group. GLIM criteria were not considered since our study was prior to the publication of the consensus. On the other hand, we consider the fact that it was a multicenter study involving a large number of patients and in real-life conditions to be a strength. It is the only study published to date with such a high number of patients with HEN and with a 6-month follow-up.

## Conclusions

The nutritional assessment to prescribe individualized HEN to each patient, together with educational measures and training in the proper use of this treatment for both patients and trainers, improves nutritional status and reduces the onset of adverse reactions.

## Supplementary Information

**Additional file 1 Table S1.** STROBE-nut: An extension of the STROBE statement for nutritional epidemiology.

**Additional file 2 Table S2. **Protein and energy requirements by diagnosed clinical condition.

## Data Availability

The datasets used and/or analysed during the current study are available from the corresponding author on reasonable request.

## Abbreviations

BMI: Body mass index
EN: Enteral nutrition
GLIM: Global leadership initiative on malnutrition
HEN: Home enteral nutrition
HP/HC: Hyperproteic/Hypercaloric
NGT: Nasogastric tube
NP/NC: Normoproteic/Normocaloric
OR: Odds ratio
PEG: Percutaneous endoscopic gastrostomy
PRG: Percutaneous radiological gastrostomy
SD: Standard deviation
Spanish NHS: The Spanish National Health System
