# Supplementary material for: Impact of nutritional and educational support on home enteral nutrition
Source: J Health Popul Nutr. 2023 May 22;42:45. doi: 10.1186/s41043-023-00384-4 (PMC10202059; doi:10.1186/s41043-023-00384-4)
Supplement: Supplementary file 2 — Additional file 2 Table S2. Protein and energy requirements by diagnosed clinical condition. [file 41043_2023_384_MOESM2_ESM.docx]

Table S2. Protein and energy requirements by diagnosed clinical condition

|  | **Condition** | | | | | |
| --- | --- | --- | --- | --- | --- | --- |
|  | **Stroke** | | **Neurodegenerative** | | **Head and neck cancer** | |
|  | **n** | **Mean± SD** | **n** | **Mean± SD** | **n** | **Mean± SD** |
| **Energy requirements (Kcal/day) (Baseline visit) *** | 89 | 1499 ± 225 | 254 | 1495 ± 252 | 42 | 1894 ± 415 |
| **Adjusted energy requirements (3-month visit) *** | 15 | 1390 ± 140 | 62 | 1433 ± 262 | 6 | 1938 ± 192 |
| **Energy requirements (Kcal/day) (6-month visit) *** | 89 | 1509 ± 195 | 250 | 1498 ± 256 | 38 | 1906 ± 399 |
| **Protein requirements (g/day) (Baseline visit) *** | 88 | 68 ± 12 | 248 | 68 ± 13 | 42 | 86 ± 18 |
| **Adjusted protein requirements (3-month visit) *** | 15 | 66 ± 9 | 62 | 68 ± 14 | 6 | 100 ± 16 |
| **Protein requirements (g/day) (6-month visit) *** | 88 | 67 ± 12 | 244 | 69 ± 13 | 38 | 86 ± 20 |
| SD: Standard Deviation, * (statistical significant difference) | | | | | | |
